# Autonomy-supportive exercise behaviors promote breast cancer survivors’ well-being

**DOI:** 10.34172/hpp.2020.60

**Published:** 2020-11-07

**Authors:** Behzad Behzadnia, Arezou Kiani, Solmaz Babaei

**Affiliations:** ^1^Department of Motor Behavior, Faculty of Physical Education and Sport Sciences, University of Tabriz, Tabriz, Iran; ^2^Neurophysiology Research Center, Urmia University of Medical Sciences, Urmia, Iran; ^3^Faculty of Humanities, University of Maragheh, Maragheh, Iran

**Keywords:** Autonomy, Breast cancer, Eudaimonia, Hedonia, Self-determination theory

## Abstract

**Background:** Grounded in self-determination theory (SDT), this pilot study aimed to test an autonomy-supportive exercise instructing style to promote hedonic (seeking comfort and pleasure) and eudaimonic (seeking to develop the best within one and pursue excellence)orientations, exercise motivation, and psychological well-being of breast cancer survivors.

**Methods:** Twenty-four breast cancer survivors were randomized in either autonomy-supportive exercise instructing style (treatment condition) or usual exercise instructing style (control condition). The study consisted of a pre-intervention session followed by 22 exercise sessions and a post-intervention session. Hedonic and eudaimonic orientations, exercise motivation, and psychological well-being (subjective vitality) measured at baseline and post-intervention sessions.

** Results:** As expected, participants in the treatment condition reported greater eudaimonic and hedonic orientations and subjective vitality compared to the participants in the control condition. The results showed no difference in exercise motivation across conditions.

**Conclusion:** We concluded that the SDT-based intervention was successful in helping breast cancer survivors increase hedonic and eudaimonic orientations and subjective vitality. Practical and theoretical implications, along with limitations and future research suggestions are discussed inside.

## Introduction

In a recent World Health Organization (WHO) report, it has shown that the overall cancer survival rate has increased because of advances in early detection and treatment worldwide, and it leads to lives many years after diagnosis.^1^ Based on WHO data, breast cancer is the most common cancer in women worldwide, which influence physical and psychological factors such as physical appearance, psychological ill-being, meaning in life, negative thinking, less activity, and low quality of life.^1-3^ Besides, when the treatment is not even lengthy patients experience difficulty returning to normal daily life.^4^ Despite these negative experiences, some oncology clinicians provide activity-related advice such as ‘take it easy’, ‘take rest’, or ‘pace yourself: do what you can but don’t push yourself’ during and after cancer treatment that might result in adverse effects in maintaining a healthy lifestyle and it can be related to worse care outcomes and decreased healthy behaviors.^5^ Therefore, this highlights the importance of research on how to engage cancer survivors in health behaviors (i.e., physical activities-related to health) and to promote their motivation and well-being. In addition, it is important to investigate cancer survivors’ well-being orientations (*hedonia* : seeking enjoyment, comfort, and pleasure, and *eudaimonia* : seeking excellence and develop the best within ones), results from health programs.^6^ In this pilot study, therefore, based on self-determination theory (SDT) approach,^7^ we aimed to examine an autonomy-supportive exercise instructing style (e.g., provide choice, and take individual’s perspectives) to enhance hedonic and eudaimonic orientations, exercise motivation, and psychological well-being in breast cancer survivors.

Recent World Cancer Report^8^ has emphasized the role of physical activity in association with 25%-30% decrease in breast cancer risk. Physical activities, generally, would be safe and has no side effect for patients during and after cancer treatment,^5^ and it would improve breast cancer survivors’ quality of life.^9^ That is, physical activities can be considered as one of the viable solutions to help breast cancer survivors maintain a normal lifestyle.^10,11^ Research has also shown that regular physical activities affect positive outcomes of physical and psychological health during and after treatment.^4,12-14^ Nonetheless, experiencing cancer and its treatment can alter the patient’s body response to physical activities (e.g., increase in death cell during chemotherapy or biochemical changes) and their quality of life.^5^ Therefore, it is important to know how physical activities can assist with maintaining physical health and general quality of life or well-being among middle-aged women.^15,16^ To do this, randomized control trials have prescribed various modes and doses of exercise for breast cancer survivors that varied from strength exercises and light physical activities (e.g., yoga) to vigorous intensity resistance and aerobic training (e.g., walking and dance).^17-19^ The intensity of exercises, generally, varied from one to five times a week and the duration of the activities varied from 20 to 60 minutes.^17^

Despite the benefits of physical activities among cancer survivors, having regular physical activity has been a challenge for cancer survivors.^20^ Research revealed that cancer survivors do not exercise sufficiently after their treatments^21^ and their participation in exercise programs is below recommended levels.^22^ Research has also emphasized that motivational regulations toward physical activity would play an important factor in determining how to enhance physical activity behaviors in cancer survivors.^23^

Motivation is a critical factor in sustaining healthy behaviors.^7^ Based on SDT, an extended theory on motivation and personality, individuals’ health and wellness is depended on the satisfaction of three basic/fundamental psychological needs. The needs are for *autonomy* (the desire to experience willingness and volition), *competence* (the desire to be effective in one’s interactions with the social environment and to seek optimal challenges), and *relatedness* (the desire to be attached and connected with others and to have a sense belonging with collectives). The satisfaction of these basic needs is crucial for positive outcomes such as behavior change, self-motivation, and well-being.^7^

A critical component of SDT is the distinction between autonomous motivation with controlled motivation and amotivation. When autonomous motivated, people are willingly engaged in an activity for personal enjoyment and interest (intrinsic motivation) and they personally valuing that activity (identified regulation). In contrast, controlled motivation refers to being motivated by internal pressures and ego-involvement (introjected regulation) and external pressures and their expectation imposed by others (external regulation). In contrast to both motivations is amotivation which refers to the absence of any motivation or no intention to behave.^7^ Research has shown that autonomous motivation is related to higher physical activity behaviors and well-being, whereas, controlled motivation and amotivation are related to unhealthy behaviors, and thus, result in disengagement and ill-being.^14,24^

SDT has explained why people choose to participate and have persistence in an activity, and how social-contexts (i.e., practitioners’ behaviors) would facilitate patients’ engagement in health-care programs. When people experience that social contexts are supportive of their autonomy and self-determination, they are more intrinsically motivated to engage in activities and result in positive outcomes. That is, practitioners’ autonomy-supportive behaviors would play a significant role in patients’ motivation and well-being.^7^ Autonomy-supportive instructions are obvious, generally, when practitioners acknowledge patients’ perspectives in their health-care programs, behave in ways that encourage patients to engage in the activities, provide positive feedback, nurturing inner motivational resources, and using non-controlling language.^25-27^ Through autonomy-supportive instructions, patients are more likely to attend treatment or recovery periods and fully involve in the activities if they believe that their practitioner respects them as an autonomous individual and cares about them. Therefore, during autonomy-supportive instructions, the role of practitioner is to facilitate treatment or recovery activities for patients by supporting their basic psychological needs.^7^ In contrast, when practitioners tend to be more controlling or low on autonomy support, use overt controlling such as threats and demanding language, and use pressuring instruction to make patients behave in prescribed ways, these can result in demotivating patients and their ill-being.^7,28^ Research has also shown that autonomy-supportive instructing style positively increased exercise motivation, well-being, and higher attendance rate in exercise programs.^26,27^

Apart from the importance of examining exercise motivation and psychological well-being, research has shown that well-being orientations (hedonia and eudaimonia) in health care programs associate with patients’ well-being.^6^ The hedonic approach reflects the view that well-being consists of maximizing subjective happiness and experience of pleasure so that to maximize hedonic well-being orientation, people should do what makes them happy and minimizing negative emotions. In contrast, the eudaimonic approach reflects optimal psychological functioning at one’s highest potential.^29^ Those who pursue a hedonic orientation in their activities are seeking to experience enjoyment, pleasure, and to take things easy, whereas, those who pursue a eudaimonic orientation are seeking to use the best within themselves, pursue excellence, and to develop and gain insight into things.^30^ Based on SDT,^7,29^ to some degree pursue both hedonic and eudaimonic orientations are important for “the good life” or optimal functioning, but when people put more importance on a eudaimonic living, they experience greater psychological well-being and fully functioning, rather than a hedonic living way.^31^ People’s chosen these orientations and behaviors may be one of the most important factors for intervening in their well-being.^32^ So, the degree to which people put importance on pursuing these orientations relate differently to their motivation toward activities and experience of well-being.^31,33^

It is important to examine both reasons *why* (i.e., motivations) patients are engaging in an activity, and reasons *what* (hedonic and eudaimonic orientations) they are seeking by doing that activity.^7^ However, to our knowledge, no research examined the effects of an autonomy-supportive exercise instructing style in enhancing hedonic and eudaimonic orientations, exercise motivation, and psychological well-being in breast cancer survivors. In this pilot study, we aimed to create an autonomy-supportive climate to motivate breast cancer survivors toward physical activities. That is, we aimed to deliver aerobic exercise training two times per week and each session length 50 to 60 minutes, in an autonomy-supportive climate compared with usual exercise training. Therefore, we hypothesized that breast cancer survivors in the treatment condition (autonomy-supportive exercise instructing style) would increase their hedonic and eudaimonic orientations, exercise motivation, and psychological well-being than would breast cancer survivors in the control condition (usual exercise instructing style).

## Materials andMethods

### Participants and procedures

Participants recruited via poster displays in hospitals, oncology centers, and via advertisements in social media (i.e., Telegram app). Hospital staff firstly contacted breast cancer survivors to recruit them and after showing their interest, consent forms were obtained from participants and their physicians to participate in this physical activity study. After that, a meeting was organized by the first author and hospital staff to introduce the program and to select patients who were met the eligibility criteria to attend the study. Eligibility criteria were based on age (middle-aged^15,16^: less than 60 years old to be able to attend the exercise programs), gender (only women were included in the study), the ability to do physical activity (qualified from their physician – free from other serious health problems), non-smoking, cancer stage (only types of I, II, and III were included in the study), types of treatment (chemotherapy, surgery, radiotherapy, and hormone therapy, or a combination of them), and all treatments needed to complete by at least two months before the beginning of the study, except hormone therapy. They were excluded if they had their regular exercise training for six months before the beginning of the study, severe or untreated psychopathology and neurologic disorders, and having two diseases/cancer simultaneously (e.g., breast cancer and diabetes).

**Figure 1:**
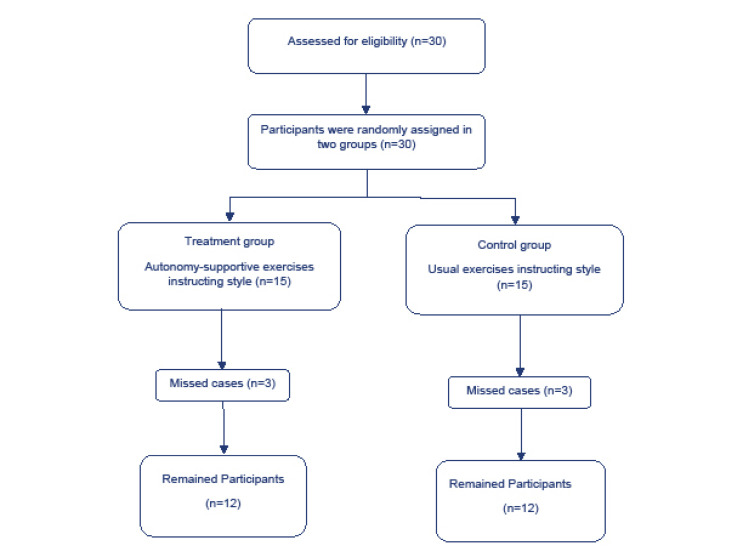
Participants’ flow chart

Thirty breast cancer survivors qualified to participate in the study, and they were randomly assigned into either autonomy-supportive exercise instructing style condition (treatment condition, n = 15) or usual exercise instructing style (control condition, *n* = 15). To randomize participants into one of the two conditions, we used the program found at https://www.random.org/sequences/. Three patients from each condition were lost to follow-up due to sickness, time constraints, travel, and not further interests in filling out the questionnaires (Figure 1). Thus, 24 participants (age range from 38 to 60 years old) (retention rate = 80%) attended in this experimental study and completed the entire study program during the spring season (late-April through mid-June). Participants received chemotherapy, surgery, radiotherapy, and hormone therapy, or a combination of them. They were also diagnosed with stage I, II, III breast cancer. All psychological variables were measured at baseline (pre test) and after the 22-sessions intervention program (post test) through self-report measures details below.

### Measures

*Orientations*

Participants’ hedonic and eudaimonic orientations were assessed using the Hedonic and Eudaimonic Motives for Activities (HEMA) developed by Huta and Ryan.^30^ The instruction to this measure was: “To what degree do you approach your physical activities with each of the following intentions, whether or not you actually achieve your aim?” The scale has five items assessing a hedonic orientation (e.g., “Seeking relaxation” and “Seeking enjoyment”), and four items assessing a eudaimonic orientation (e.g., “Seeking to pursue excellence or a personal ideal”). The items were rated from 1 (*not at all* ) to 7 (*very much* ). The HEMA has previously translated and validated into Persian by Behzadnia and Ryan.^31^

*Exercise Motivation*

Participants’ exercise motivation was measured through the *Behavioral Regulation in Exercise Questionnaire* (BREQ-2).^34^ The instruction to this measure was “I engage in exercise because …” Sample items for each of the motivational regulations are: “I enjoy my exercise sessions” (intrinsic motivation), “I value the benefits of exercise” (identified regulation), “I don’t see the point in exercising” (introjected regulation), “I exercise because other people say I should” (external regulation), and “I think exercising is a waste of time” (amotivation). The items were rated from 0 (*not true for me* ) to 4 (*very true for me* ). Previous research reported acceptable internal reliability of the BREQ-2 in Iranian samples.^35^ Based on research by Ryan and Connell,^36^ we computed a Relative Autonomy Index^37^ that represents the overall self-determination. To form the RAI, the following formula to combine subscales were used: 3 X Intrinsic + 2 X Identified - Introjected - 2 X External - 3 X Amotivation. Higher scores in the RAI indicate higher autonomous motivation, whereas lower scores indicate less autonomous motivation. The maximum possible score of the RAI is 20 and the minimum is -24.

*Well-being*

To assess well-being the five-item version of the scale of the *subjective vitality* (e.g., “I feel alive and vital” and “I look forward to each new day”) was used.^38^ Participants responded to the stem, “To what degree do you typically feel each of the following …” Responses ranged from 1 (*not at all true* ) to 7 (*very much true* ). Previous research reported acceptable internal reliability of this scale in an Iranian sample.^31^

*Intervention*

According to the guidelines provided by the American Cancer Society^4^ and previous research,^39,40^ exercise programs were created for both conditions. Along with exercise programs, participants in the treatment condition were received an autonomy-supportive instructing style. That is, the instructor in the treatment condition behaved based on the autonomy-supportive instructing style^7^ (see Table 1). The instructions were adapted from previous research in the area of physical activities^41-43^ and health^26,27^ programs. The construction of the autonomy-supportive climate was based on several important points, (*a* ) to create a climate to support participants’ autonomy need satisfaction, (*b* ) to enhance autonomous motivation toward activities (e.g., physical activity), and (*c* ) to enhance awareness regarding their choices and behaviors. The instructor, for example, in the exercise sessions tried to take participants’ perspectives on how to do exercises, supported their decisions, use non-controlling languages when asked participants to do exercises, and provided positive feedback regarding participants’ performance.

Participants in both conditions were blinded to the study goals, and they were only informed that the research aimed to examine their psychological states in general. Participants in the control condition did not receive autonomy-supportive intervention program aimed at the treatment condition.

### Exercise program

Participants attended exercise programs three times per week, each session length 50-60 minutes as follows: initial cardiovascular activities and stretch or flexibility exercises as warm-up training (15 minutes), main aerobic dance training (20-30 minutes), and cool down and stretch training program (10 minutes). Polar heart rate monitors during exercise sessions was used to maintain the goal of 55%-60% of heart rate reserve (HRR). Exercise programs progressed gradually based on the participants’ physical fitness. That is, the first 10-session exercises included basic aerobic dance training (55% HRR, about 20 minutes), and the rest of the 12-session exercises included progressive training from basic-level to moderate-level aerobic dance training (60% HRR, about 30 minutes). Exercise sessions were group-based either in treatment or control conditions.

Exercise sessions at both treatment and control conditions were supervised by the research staff members (i.e., psychologist [expert in SDT] and sport physiologist). The systematic observation of training sessions in terms of exercise programs and autonomy-supportive instructions (treatment condition vs. control condition) carried out by research staff in each condition and they observed and provided feedbacks on some sessions randomly in each condition.

### Data analysis

Analyses were carried out with SPSS version 22.0 for Windows. Data were firstly screened for missing values, the presence of statistical outliers, and normality. Means, standard deviation (SD), internal reliability (*α* ), at baseline (pre test) and post (post test) exercise sessions in both treatment (autonomy support) and control (non-autonomy support, usual training) conditions are shown in Table 2. Internal consistency of measures was computed through Cronbach’s alpha, that presents in Table 2.

To examine the study hypothesis, we examined the effect of either an autonomy-supportive exercise instructing style or usual exercise instructing style on hedonic and eudaimonic orientations, exercise motivation, and subjective vitality. To assess the effects of the autonomy-supportive intervention on participants’ hedonic orientation, exercise motivation, and subjective vitality, we conducted three 2 (time of assessment) × 2 (intervention: autonomy support and usual exercise instructions) repeated measure ANOVAs, (one for each variable). To assess the effects of the autonomy-supportive intervention on participants’ eudaimonic orientation, an analysis of covariance (ANCOVA) was conducted. Because of the small sample size per group (n ~ 12), it was necessary to adjust the statistically significant level from P < .05 to P < 0.10.^44^ Giving the small sample size in the current study (n ~ 12 per group), and the high probability of making type 2 error, a significance level with of P < 0.10 conducted in the principal analyses.

## Results

Descriptive statistics in two time points in both conditions are presented in Table 2. Primary analyses showed that participants’ age was not correlated with the study variables (see Table 3). Next, the multivariate analysis of variance (MANOVA) was conducted to examine the mean differences for types of treatment (chemotherapy, surgery, radiotherapy, hormone therapy, or a combination of them) and stages of diagnosis (stages of I, II, and III breast cancer). The results showed that participants were not different based on their type of treatment (*P* = ns) and stage of diagnosis (*P* = ns) on the study variables.

The mean value of measures did not differ at baseline between treatment and control conditions, except for the mean values of the eudaimonic orientation (*t* = 3.36, *P* = 0.003, mean diff = 1.45) that was higher in the control condition compared to the treatment condition. Giving this finding, we included eudaimonic orientation measure at baseline as a covariate in the main analysis. Participants reported no major health problems in both conditions during exercises. Moreover, participants in the treatment condition showed satisfaction/interest in their intention to continue exercise programs after the program end.

For participants’ *eudaimonic orientation* , the results of ANCOVA showed that there was a significant difference between the treatment and control conditions at post test after controlling for participants’ eudaimonic orientation at baseline (pre test), *F* (1, 21) = 14.59, *P* = 0.001, ƞ_p_^2^= 0.41. Participants in the treatment condition showed higher eudaimonic orientation than participants in the control condition at post test. Moreover, pairwise comparisons showed that eudaimonic orientation increased significantly for the participants in the treatment condition from pre to post test ( P < 0.001, *d* = 1.52, 95% CI [0.96, 2.24]), whereas it decreased for the participants in the control condition from pre to post test (*P* = 0.019, *d* = 0.88, 95% CI [-0.15, -1.39]).

For participants’ *hedonic orientation* , the results of repeated measure ANOVA showed that only the main effect for interaction of time × condition was significant, *F* (1, 22) = 10.56, *P* = 0.004, ƞ_p_^2^= 0.32, and the main effects for time and condition were not significant. Hedonic orientation increased for the participants in the treatment condition from pre to post test (*P* = 0.048, *d* = 0.75, 95% CI [0.01, 1.19]), whereas it decreased for the participants in the control condition from pre to post test (*P* = 0.035, *d* = 0.91, 95% CI [-1.72, -0.08]). Moreover, participants in the treatment condition showed significantly higher hedonic orientation than participants in the control condition at post test (*p* = 0.022, *d* = 1.02, 95% CI [0.16, 1.81]).

For participants’ *exercise motivation* , the results of repeated measure ANOVA showed that none of the main effect for the interaction of time × condition, the main effect for time, and the main effect for condition were significant.

For participants’ *subjective vitality* , the results of repeated measure ANOVA showed that the main effect for time, *F* (1, 22) = 5.83, *P* = 0.025, ƞ_p_^2^= 0.21, and the main effect for interaction of time × condition, *F* (1, 22) = 12.09, *P* = 0.002, ƞ_p_^2^= 0.36, were significant, but the main effect for condition was not significant. Subjective vitality increased for the participants in the treatment condition from pre to post test (*P* = 0.009, *d* = 1.38, 95% CI [.35, 1.92]), whereas it remained unchanged for the participants in the control condition from pre to post test. Moreover, participants in the treatment condition showed significantly higher subjective vitality than participants in the control condition at post test (*P* = 0.016, *d* = 1.06, 95% CI [0.14, 1.20]).

## Discussion

When cancer treatment ends, getting back to normal life is difficult as survivors have been experienced psychological ill-being and less hope of living longer.^4^ According to SDT recommendations,^7^ we aimed to create a need supportive environment for breast cancer survivors to promote their well-being orientations, exercise motivation, and psychological well-being. Consistent with our expectations, generally, the effect of the intervention was successful. The results showed that participants in the treatment condition (autonomy-supportive exercise instructing style) increased their hedonic and eudaimonic orientations and psychological well-being relative to the participants in the control condition (usual exercise instructing style).

This study was the first to explore the role of autonomy-supportive behaviors on breast cancer survivors’ well-being orientations and well-being experience. In line with SDT,^7^ the results showed that supporting breast cancer survivors’ autonomy and self-determination are important in promoting their hedonic and eudaimonic orientations and subjective vitality, compared to usual exercise instructing programs that emphasize is not enhancing self-determination in the activities. In other words, behaving in autonomous ways and supporting autonomy are important for optimal well-being. In this study, we found that when the practitioner respect breast cancer survivors as autonomous individuals in doing their exercises, support their choices and decision making, they enjoy the activities and more intrinsically develop their skills and seek to use the best within themselves through exercise programs. Therefore, findings provided support for SDT, and provided promising evidence that physical activities through autonomy-supportive instructing style may lead to greater hedonic and eudaimonic orientations in breast cancer survivors.

We also found that autonomy-supportive behaviors positively promoted breast cancer survivors’ subjective vitality. This finding, generally, is of relevance as previous research has shown that physical activities were positively promoted breast cancer survivors’ psychological well-being.^45^ It is important to note that, behaving with cancer survivors in an autonomy-supportive way has manifold implications. Cancer survivors need to feel care from others, feeling connected and warmth with others, providing them with positive feedback in doing their activities, acknowledge their negative feelings, and talk with them through encouraging languages – because these behaviors not also are important for their targeted behaviors, but also are important to giving them more energy to move and to regulate their emotions. Thus, creating a need supportive climate would produce breast cancer survivors’ positive outcomes to manage their health behaviors.^7^

Among the most conceptualization of well-being or good life is *subjective vitality* – the concept that is related to feeling energized and excited, which is also counter with ego depletion. Research has also shown that higher subjective vitality was related to healthier physiological functioning.^46^ Within SDT, it has shown that subjective vitality increases by the activities that satisfy basic needs (i.e., autonomy support), and it has also related to higher intention to do activities.^47^ In this study, we provided support for this proposition in breast cancer survivors.

### Practical implications

The present study suggests that a need supportive climate is effective at promoting breast cancer survivors’ hedonic and eudaimonic orientations and their well-being. Therefore, the information provided in this study can inform practitioners (or exercise training consultants), and physicians, that supporting breast cancer survivors’ choices and respecting them as autonomous individuals in physical activity environments can promote their positive functions of well-being orientations and well-being experience.

Practitioners and future interventions would focus on enhancing basic psychological needs and well-being orientations alongside physical activity programs in breast cancer survivors. To do that, for example, future research can examine how supporting all three basic need support, autonomy support, competence support (e.g., providing survivors with informational feedback) and relatedness support (e.g., showing survivors that they are interested in and care for what they do) alongside physical activity programs attribute to well-being and exercise motivations. In addition, future research can examine more positive psychological well-being elements (e.g., positive affect, life satisfaction, and meaning in life) and ill-being elements (e.g., depression, anxiety, and negative affect), as well as physical well-being indicators such as immunological functioning. Such experimental research would enable researchers to examine whether a need supportive training style would increase orientations toward well-being, exercise motivation, and well-being experiences as well as reduce amotivation and ill-being.

Aside from positive effects of the intervention, the results found no significant change in breast cancer survivors’ exercise motivation. A low sample of participants might cause this. Because of the relatively small sample size, we could not measure different types of motivational regulations (i.e., intrinsic motivation, identified, introjected, and external regulations, and amotivation), therefore future research needs to replicate these findings with a large number of breast cancer survivors. It is also possible that participants’ motivational regulations needed longer times to change, as the SDT claims that internalization of motivations needs time.^7^ For example, identified and introjected regulations may need longer times to internalize the value and importance of exercise by cancer survivors. Therefore, longer experimental and longitudinal studies would provide useful knowledge concerning breast cancer survivors’ exercise motivation.

Despite the limitations of this study, low sample size and only examined breast cancer survivors, the present study provides an important role of autonomy-supportive behaviors in promoting well-being in breast cancer survivors. Further research that examines cancer survivor samples may also consider other important points deriving from this study like measuring more time points to detect changes over time, and investigate other kinds of cancer samples (e.g., prostate) during and after treatments. Also, examining other variables such as individual differences in causality orientations that would interact with social-context effects (i.e., practitioners’ autonomy support) might provide important knowledge regarding the effectiveness of the intervention.^7^ Moreover, future research may also benefit from a non-exercise control group, to see how variables employed in this study would change over time without a regular activity.

## Conclusion

The present study showed that creating an autonomy-supportive environment is effective in enhancing breast cancer survivors’ hedonic and eudaimonic orientations and psychological well-being. Practitioners and physicians can use this intervention to fill the gap between expectations and health care programs, by supporting breast cancer survivors’ self-determination, providing with the choice, and respecting them as autonomous individuals in their physical activity programs.

## Acknowledgments

The authors thank Omid Cancer Hospital for helping to recruit breast cancer survivors.

## Funding

This study was partly funded by grant 1393125 from Neurophysiology Research Center, Urmia University of Medical Sciences, Urmia, Iran.

## Competing interests

The authors have no conflicts of interest relevant to this article.

## Ethical approval

All the study procedures reviewed and approved by the Medical Ethics Committee of the University of Medical Sciences, Ministry of Health, I.R. Iran, that is according to guidelines of the Declaration of Helsinki.

## Authors’ contributions

All authors contributed to the study equally.

**Table 1:** Instructions in the treatment condition (autonomy-supportive interventions)

**Instructional behaviors**	**Instructions**
Provided choice	The practitioners provided with cancer survivors’ choice and supported their decisions on how to do exercise; given time to work (exercise: strengthen – warm up – cool down); displayed patience; and organized the exercise in a way that cancer survivors preferred.
Provided positive feedback and meaningful rationale	The practitioner provided meaningful rationale on why exercise is important for them (e.g., the importance of exercise in their psychological and physical health); the practitioner sometimes simplified exercises (the impact of aerobic exercises) for cancer survivors (when the practitioners felt that they are bored or tired; and provided positive feedback related to their abilities to do exercise.
Perspective-taking	The practitioners allowed cancer survivors interest and preferences to guide their activities (perspective-taking emphatic statements); listened to cancer survivors’ opinions; spend time talking with them and made a good relationship with them and displayed care about them; respected cancer survivors as autonomous individuals; took their suggestion regarding when they would like to do exercise and dance; and how they would prefer to do their exercises.
Informational and encouraging language	Used informational and nondirective language to conduct class time; encouraged cancer survivors by some positive statements (e.g., “you can” rather than “you have to”) to continue exercises and participating in exercise sessions; and encouraged their happy activities during class times.
Acknowledged cancer survivors’ feelings	The practitioners asked what cancer survivors want or desire; acknowledge cancer survivors’ feelings about exercises; avoided controlling behaviors and ego-involvements as well as accepted their negative affect expressions; and listened and asked questions related to exercise programs.

**Table 2:** Descriptive statistics in both conditions on pre and post test sessions

	**α**	**Treatment (Autonomy support)**				**Control (Usual)**			
		**Pre-test**		**Post-test**		**Pre-test**		**Post-test**	
		**Mean**	**SD**	**Mean**	**SD**	**Mean**	**SD**	**Mean**	**SD**
Hedonic orientation	0.63	5.37	.85	5.97	0.73	5.88	0.77	4.98	1.17
*Eudaimonic* orientation	0.75	4.72	1.22	6.31	0.84	6.17	0.87	5.40	0.88
RAI	0.77	10.44	5.41	11.90	2.51	10.67	4.39	8.71	4.42
Subjective vitality	0.76	5.36	1.01	6.50	0	6.04	0.85	5.83	0.67

*Note* : RAI, Relative Autonomy Index.

**Table 3:** Correlation among treatment condition and the study variables in two waves

		**1**	**2**	**3**	**4**	**5**	**6**	**7**	**8**	**9**
1	Experimental condition	1								
	*Time 1*									
2	Hedonic orientation	-0.31	1							
3	Eudaimonic orientation	-0.58^**^	0.24	1						
4	Subjective vitality	-0.35	0.38	0.17	1					
5	RAI	-0.03	-0.07	0.03	-0.01	1				
	*Time 2*									
6	Hedonic orientation	0.47^*^	0.04	-0.21	-0.17	0.08	1			
7	Eudaimonic orientation	0.49^*^	-0.10	0.06	-0.28	-0.06	0.62^**^	1		
8	Subjective vitality	0.49^*^	0.15	-0.26	0.09	0.01	0.54^**^	0.55^**^	1	
9	RAI	0.42^*^	0.07	-0.15	-0.45^*^	-0.12	0.64^**^	0.58^**^	0.29	1
10	Age	-0.21	0.35	0.05	-0.05	-0.19	0.16	-0.15	-0.12	0.16

*Note* : RAI, Relative Autonomy Index ^*^ P < 0.05, ^**^ P < 0.01.
